# HOPE for Hypermobile Ehlers–Danlos Syndrome (hEDS) and Hypermobility Spectrum Disorder (HSD)—A Pilot Randomised Controlled Trial of Feasibility, Acceptability and Appropriateness

**DOI:** 10.1002/ejp.70030

**Published:** 2025-04-29

**Authors:** Min Tze Chew, Emre Ilhan, Leslie L. Nicholson, Sarah Kobayashi, Verity Pacey, Alan Hakim, Cliffton Chan

**Affiliations:** ^1^ Department of Health Sciences, Faculty of Medicine, Health and Human Sciences Macquarie University North Ryde New South Wales Australia; ^2^ Faculty of Medicine and Health, Kolling Institute University of Sydney St Leonards New South Wales Australia; ^3^ The Ehlers‐Danlos Society, The Ehlers‐Danlos Society – Europe London UK

## Abstract

**Background:**

Feasibility and effectiveness of online pain management programs for chronic widespread pain conditions have been shown; yet, there are no empirically evaluated programs for hypermobile Ehlers–Danlos syndrome (hEDS) or hypermobility spectrum disorder (HSD). Bridging this gap, the Hypermobile Online Pain managemEnt (HOPE) program was developed with stakeholder input to be pilot tested.

**Methods:**

We conducted a randomised controlled trial of the HOPE program to assess Feasibility of Intervention Measure (FIM), Acceptability of Intervention Measure (AIM) and Intervention Appropriateness Measure (IAM) and eight clinical effectiveness outcomes. Intervention participants were given 8 weeks to complete 12 self‐paced modules, while control participants continued treatment as usual. Participants were assessed at baseline, post‐treatment and 3 months post‐treatment using online surveys.

**Results:**

Seventy‐two participants were recruited and randomised. Two control participants withdrew before starting, leaving 34 controls and 36 intervention participants for analysis. In all, 81%–91% agreed/completely agreed that the HOPE program was feasible with mean FIM score of 4.3/5 (SD 0.7), 62%–76% agreed/completely agreed that it was acceptable with mean AIM score of 3.9/5 (SD 0.96) and 67%–76% agreed/completely agreed that it was appropriate with mean IAM score of 4.0/5 (SD 0.9). Only two clinical effectiveness outcomes were significantly improved in the intervention group, with moderate and small effect sizes in worst pain intensity (Cohen's *d* = 0.63) and impact of hypermobility (Cohen's *d* = 0.32) at 3 months post‐intervention, respectively.

**Conclusion:**

The HOPE program seemed feasible, acceptable and appropriate, with preliminary improvements in worst pain intensity and impact of hypermobility. Our findings warrant a fully powered trial to further explore the clinical effectiveness of this online pain intervention.

**Significance Statement:**

Pain is of high concern among people with hypermobile Ehlers–Danlos syndrome (hEDS) or hypermobility spectrum disorder (HSD), yet there are limited online pain management resources for them. This pilot trial of the novel Hypermobile Online Pain managemEnt (HOPE) program is important in guiding the creation of evidence‐based and stakeholder‐relevant online resources. Promising results suggest the importance of further refinement and retesting its effectiveness before wider implementation among the hEDS/HSD population so as to empower them in pain self‐management.

## 1 Introduction

Ehlers–Danlos syndromes (EDS) are heritable disorders of connective tissue with fourteen subtypes; thirteen are officially classified and included in the 2017 International Classification for the Ehlers–Danlos syndromes (Blackburn et al. 2018; Malfait et al. 2017, 2020). Hypermobile Ehlers–Danlos syndrome (hEDS) is the most common, followed by hypermobility spectrum disorder (HSD), both displaying significant clinical similarities (Castori and Hakim 2017). The reported prevalence of all EDS and HSD combined is 1 in 500, with hEDS and HSD accounting for over 95% of all cases (Demmler et al. 2019). These conditions can affect the musculoskeletal, neurological, gastrointestinal, cardiovascular, ocular, oro‐dental, airway and urogenital systems (Tinkle et al. 2017).

Pain is one of the most common symptoms in this population, with a reported prevalence of 86%–98% (Teran‐Wodzinski and Kumar 2023; Wasim et al. 2019). The pain mechanisms are complex and highly challenging to diagnose and treat (Carroll 2023; Gensemer et al. 2021). There are likely concurrent biological mechanisms in each pain experience. For example, nociceptive mechanisms dominate in acute subluxations and dislocations. Recurrent injury and prolonged pain experiences may cause central sensitisation and nociplastic pain (Di Stefano et al. 2016). Small fibre nerve damage and entrapments contribute to neuropathic pain (Fernandez et al. 2022; Igharo et al. 2023). Living with this chronic and painful condition can be psychologically burdensome (Cederlöf et al. 2016; De Baets et al. 2022). Socially, this population is often misunderstood, isolated and poorly managed medically (Anderson and Lane 2021; Halverson, Penwell, et al. 2023). These challenges contribute to ongoing pain and disability (van Meulenbroek et al. 2021). Hence, the biopsychosocial model of pain may be well suited to account for this population.

There is a need for evidence‐based and stakeholder‐informed pain management options encompassing the unique pain mechanisms and experiences of those with hEDS/HSD. Research recommendations suggest incorporating a multidisciplinary, biopsychosocial model (Baeza‐Velasco et al. 2019), whilst people with hEDS/HSD want guidance and support (Bennett et al. 2021). Several face‐to‐face multidisciplinary pain management programs for hEDS/HSD have been trialled with promising results but require significant resources, participant travel or inpatient stay (Chaleat‐Valayer et al. 2019; Hakimi et al. 2023). These can be costly, time intensive and inaccessible to those who have unpredictable and activity‐limiting symptoms, financial difficulties or no access to EDS‐aware facilities (Estrella and Frazier 2024). Online programs may pose a solution. They have been implemented successfully for generic use (Dear et al. 2018; Deegan et al. 2023) and specific conditions like fibromyalgia (Friesen et al. 2017). To date, there are no empirically tested, online, asynchronous pain management programs utilising the biopsychosocial approach for hEDS/HSD (Chew et al. 2023).

The Hypermobile Online Pain managEment (HOPE) program is the first such program developed based on a modified Delphi study of stakeholders: people with hEDS/HSD and healthcare professionals. The primary aim of this pilot study was to assess its feasibility, acceptability and appropriateness; the secondary aim was to assess its effectiveness. The results will guide the need for and/or conducting of future trials of the program or similar interventions for this cohort.

## 2 Materials and Methods

### 2.1 Study Design

This study was prospectively registered with the Australian New Zealand Clinical Trials Registry (ACTRN12623001323617) and follows the Consolidated Standards of Reporting Trials Statements (CONSORT)—Pilot and Feasibility Trials (Eldridge et al. 2016) (see Appendix S1 for checklist). This study was a 2‐arm, parallel‐design randomised controlled trial carried out in Australia and approved by Macquarie University's Human Research Ethics Committee (520231630154261).

### 2.2 Participants

**FIGURE 1:**
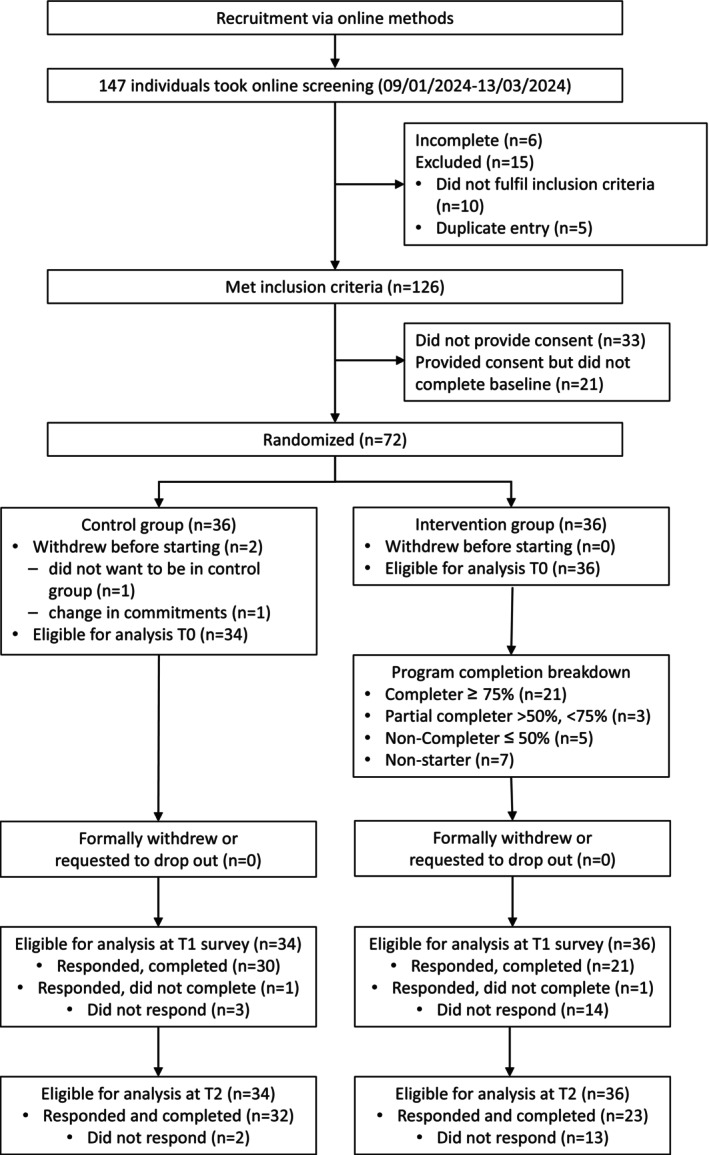
CONSORT flow chart.

Recruitment and eligibility screening were performed from January to mid‐March 2024 (see Figure 1). We recruited participants from the Ehlers–Danlos Society social media pages, major Australian hEDS/HSD social media groups and emailing persons‐in‐charge of hEDS/HSD mailing lists, publicly listed hEDS/HSD support groups, private clinics or hospitals that treat people with these conditions. We screened participants and sought consent online via the institution's Research Electronic Data Capture (REDCap) website (Harris et al. 2019, 2009).

### 2.3 Eligibility Criteria

Individuals were included in this study if they were 18 years and over; Australian residents; diagnosed by a medical doctor as having hEDS or HSD (previously known as joint hypermobility syndrome [JHS] or Ehlers–Danlos syndrome—hypermobile type [EDS‐HT]), experiencing pain for more than 3 months, proficient in English to engage with the program, able to access a desktop/laptop computer and internet over the course of the program, and on stable dosage and type of pain medication and stable medical and/or allied health interventions for pain before being recruited. Individuals were excluded if they had severe depression (Patient Health Questionnaire 9‐item [PHQ‐9] score ≥ 20) (Kroenke et al. 2001), significant suicidal ideation (PHQ‐9 score > 2 on item 9) or co‐existing medical conditions causing pain unlikely associated with hEDS/HSD such as active cancer or active joint inflammatory conditions.

### 2.4 Randomization, Blinding and Study Enrolment

Two authors were involved in the recruitment and randomization stages to maintain concealed group allocation for the authors involved in data analysis. The randomization sequence was generated by one author (EI) not involved in recruitment, using an online sequence generator with an allocation ratio of 1:1 and block stratification of 2, 4 and 6. This sequence was kept at a central location and revealed to the author (MTC) involved in recruitment and enrolment via email. All timestamps of allocation emails (between researchers MTC and EI, and between MTC and participants) were recorded for tracing and transparency purposes. This process allowed all authors to be blinded to participant results. Participants could not be blinded to group allocation in this study.

### 2.5 Intervention: HOPE Program

Participants in the intervention group took part in HOPE, an 8‐week asynchronous online pain management program that consisted of 12 modules. Participants were encouraged to complete two modules per week, with two extra weeks to enable catch up due to the dynamic nature of their condition. Each module was developed to take 30–45 min to complete. The HOPE program incorporates components of pain neuroscience, condition‐specific education and reflective questions. The aims of the content were to provide education about pain using the biopsychosocial model to help the individual ‘make sense of their pain’. The content was followed by optional reflective questions that provided time and space for participants to reflect on their own cognition and emotions related to their pain, and to consider and action their own lifestyle and behaviour changes as part of creating their own pain self‐management plan. The rationale for using this content and reflection questions is based on some of the principles of cognitive functional therapy, which has been shown in a recent large‐scale study to improve pain‐related activity limitations in chronic low back pain (Kent et al. 2019). The reflective questions provided participants with an opportunity to apply and engage with each module's content. For example, in Module 1, which covers the biopsychosocial approach to pain, participants were prompted to reflect on and record their individual pain experiences and beliefs. In subsequent modules, participants were then guided by further reflection questions on how they could incorporate their biopsychosocial factors into their pain action plan. These questions enable participants to put lessons into action, creating and implementing their own individualised actions plans to self‐manage their pain. For a description of each module, please refer to Appendix S2. The HOPE website was hosted on Macquarie University's Wordpress (WordPress.com) platform using the LearnDash (https://www.learndash.com) Learning Management System plugin. It was password protected and managed by the research group and the Macquarie University Research Data and Software team. Participants were provided with individual login details and had access to the course for the 8 weeks only. Participants in the intervention group were to continue usual treatment and received three reminder emails to complete the program over the 8‐week intervention period.

Participants in the control group were asked to continue their treatment as usual. They were not given access to the HOPE program.

### 2.6 Demographic and Clinical Characteristics

Participant demographics including age, gender identity, relationship status, pregnancy status, highest education level and employment status were collected via online surveys.

### 2.7 Outcome Measures

#### 2.7.1 Primary Outcomes: Feasibility, Acceptability and Appropriateness

The primary outcomes for this study were the feasibility, acceptability and appropriateness of the HOPE program. These outcomes were three of the eight implementation measures defined by Proctor and colleagues (Proctor et al. 2011) recommended for the early stages of implementation trials. The authors defined feasibility as the extent of success or failure of an intervention in each setting, and this was measured here using the Feasibility of Intervention Measure (FIM) (Weiner et al. 2017). Additionally, we collected information about the number of visits on each page of the program using Google Analytics (https://developers.google.com/analytics), as well as information about the percentage completion for each participant using LearnDash analytics. The program format was run in a linear fashion where participants completed one module before moving on to the next. Program completion was defined a priori as completing ≥ 75% of the modules (i.e., up to Module 9, see Appendix S2), while non‐completion was defined as ≤ 50% (i.e., up to Module 6). Completion rates in between were considered as partial completion. Study adherence was collected by recording the number of participants who completed the surveys. We also looked at whether our intervention implementation and data collection methods were appropriate and if they were collected at time points matching our protocol.

Acceptability was defined as stakeholders' (both provider and consumer) perception of whether the intervention was ‘agreeable, palatable, or satisfactory’ (Proctor et al. 2011), measured using the Acceptability of Intervention Measure (AIM) (Weiner et al. 2017). Appropriateness was defined as the ‘fit, relevance or compatibility’ of the intervention for a given setting, provider or consumer (Proctor et al. 2011), measured using the Intervention Appropriateness Measure (IAM) (Weiner et al. 2017).

There were four questions for each of the FIM, AIM and IAM surveys. Participants were asked to rate their level of agreement to each item on a 1–5 Likert scale, with 1 being *completely disagree* to 5 being *completely agree*. Psychometric testing of these measures has been performed; reliability and validity are good, with test–retest reliability coefficients ranging from 0.73 to 0.88 (Koo and Li 2016). Internal consistency was good to excellent (intraclass coefficient = 0.82 for acceptability, 0.94 for appropriateness and 0.87 for feasibility) (Bobak et al. 2018), and similarly, test–retest validity was reported as acceptable (Pearson correlation coefficients = 0.8 for acceptability, 0.73 for appropriateness and 0.88 for feasibility) (Weiner et al. 2017). Content validity was also reported, with a comparative fit index of 0.96 (Weiner et al. 2017). No cut‐off scores are available for the FIM, AIM and IAM, but recent studies using these outcomes used a mean cut‐off of > 4 for each survey or > 15 for the summed mean to define high scores (Li et al. 2024; Smeekens et al. 2024). The FIM, AIM and IAM were analysed for participants who completed the online surveys at post‐intervention (T1).

#### 2.7.2 Secondary Outcomes: Effectiveness of the HOPE Programme

Secondary outcomes, used to assess the effectiveness of the HOPE program, were collected at three time points: baseline (T0), post‐intervention (T1) and 3‐months post‐intervention (T2). Outcome measures were based on recommendations from the Initiative on Methods, Measurements and Pain Assessment in Clinical Trials (IMMPACT) (Dworkin et al. 2005) and the electronic Persistent Pain Outcomes Collaboration (ePPOC) (Nicholas et al. 2019). These included

- Brief Pain Inventory—Short form (BPI‐SF) (Cleeland and Ryan 1994). This 15‐item self‐report measure evaluates interventions in chronic pain trials (Poquet and Lin 2016), with internal consistency and test–retest reliability established for non‐cancer pain (Mendoza et al. 2006). Two pain constructs are measured, intensity (at worst, least, average pain and pain right now) and interference, using a 0–10 numeric rating scale. Interference is reported as a summed score ranging from 0 to 70. Higher scores indicate higher pain intensity and interference.
- Bristol Impact of Hypermobility (BIOH) questionnaire (Palmer, Cramp, et al. 2017). The BIOH measures the impact of joint hypermobility on a person's day‐to‐day life. Its test–retest reliability and validity have been established in adults with JHS (Palmer, Cramp, et al. 2017; Palmer, Manns, et al. 2017). There are 55 items covering pain, fatigue, physical function and mood domains, rated on a 5‐ or 10‐point Likert scale. The maximum score is 360, with higher scores reflecting greater severity of the impact of hypermobility.
- Depression, Anxiety and Stress Scale (DASS‐21) (Lovibond and Lovibond 1995). This shortened version of the DASS comprises 21 questions, seven for each domain. The DASS‐21 has shown satisfactory reliability and validity in clinical and non‐clinical groups (Antony et al. 1998). Participants are asked to rate their response on a 4‐point Likert scale, and their total scores are multiplied by 2 to enable interpretation based on the original DASS (Henry and Crawford 2005). Scores range from 0 to 42 for each domain, with higher scores reflecting greater depression, anxiety or stress.
- Patient Self‐Efficacy Questionnaire (PSEQ) (Nicholas 2007). The PSEQ measures confidence in one's ability to perform tasks despite pain, with satisfactory reliability and validity established in chronic pain populations (Nicholas 2007). There are 10 questions rating confidence in performing certain tasks using a 7‐point Likert scale. Scores range from 0 to 60, with higher scores indicating higher confidence.
- Patient Global Impression of Change (PGIC) (Guy 1976). A single question rates overall change since starting the study using a 7‐point Likert scale. Higher scores indicate a worsening of health status (Guy 1976).
- Change in pain medication and/or other pain treatment use, such as visits to health professionals during the intervention period. This was asked using two questions: (i) has your usage of pain medication(s) increased, decreased or stayed the same and (ii) have you had to see your health professionals(s) for your pain more, less or the same?
- Adverse event reporting. Participants were asked 3 questions: whether they experienced any (1) life‐threatening injuries, (2) injuries resulting in hospitalisation, and (3) new injuries causing persistent or significant disability or incapacity during the intervention period. These questions are based on the National Health and Medical Research Council definitions of adverse events and reactions (National Health and Medical Research Council 2016).
- Minimal clinically‐important difference (MCID) based on the secondary outcome measures: BPI‐SF, BIOH, DASS‐21, PSEQ. For each of these secondary outcome measures, intervention participants were asked, ‘Considering what we have told you about this HOPE program and what it involves, how much improvement in the following outcomes would you expect for this intervention to be worthwhile for you’. This allowed us to use a within‐person, anchor‐based approach to calculate each participant's MCID as a percentage (Dworkin et al. 2008) to determine how many participants met or exceeded their expected MCID post‐intervention.

Of these secondary measures, change in pain medication and/or other pain treatment use, PGIC and adverse events were measured at post‐intervention (T1) and 3 months post‐intervention (T2); MCID was measured at baseline (T0), and all others were measured at T0, T1 and T2.

### 2.8 Sample Size

As this was a pilot trial with primary aims of determining feasibility, acceptability and appropriateness, we did not perform a sample‐size calculation. We planned to recruit 70 participants to allow for study attrition, which has been reported as up to 28% for randomised controlled trials in this population (Reychler et al. 2021). This would allow for at least 50 participants for analysis. No interim analyses or stopping guidelines were used.

### 2.9 Data Analysis

All data analyses were performed using IBM SPSS Statistics for Mac (version 29.0) or Microsoft Excel (version 16.91) by researchers blinded to group allocation. Descriptive statistics were used to summarise and report participant baseline demographics and primary outcomes as means (SD), continuous data and frequency (percentages) for categorical data. Study adherence rates and programme completion rates were reported as frequencies (percentages). Independent samples *t*‐tests or chi‐square tests were used to compare baseline characteristics of the intervention and control groups for parametric and non‐parametric data, respectively. Statistical significance was set at *p* value < 0.05. Complete case analysis was used to evaluate the FIM, AIM and IAM, and summarised using frequency (percentages) for categorical data or mean (SD) for continuous data. Both approaches to summarising and reporting the FIM, AIM and IAM are typically used in the literature (Fioratti et al. 2022; Kamran et al. 2024; Reilly et al. 2021). Therefore, we have chosen to present both, and these measures were analysed using a complete case analysis due to the lack of recommendations for analysing missing data for FIM, AIM and IAM.

Secondary outcomes were analysed according to the intention‐to‐treat principle using a general linear model (ANCOVA) to determine between‐group differences at post‐intervention and 3 months post‐intervention. Models were adjusted based on baseline outcomes, included in the model as covariates. The assumptions of ANCOVA were satisfied. Given the pilot nature of this trial, the measure of uncertainty was set a priori at an 85% confidence level (Lee et al. 2014), and a *p* value of < 0.15 was considered statistically significant. For each secondary outcome, estimated effect sizes were reported as Cohen's *d*, with a value of 0.2 indicating a small effect, 0.5 as a medium effect and 0.8 as a large effect (Cohen 2013). Unadjusted descriptive statistics for each secondary outcome, at each time point, were calculated and reported. The frequency (percentage) of individuals who met or exceeded their baseline MCID at post‐intervention and 3 months post‐intervention was calculated. This was achieved by comparing the percentage change expected at baseline to the actual change experienced by the participant on the relevant outcomes. For example, if a participant expected a 30% improvement in pain intensity, then the percent change in pain intensity between baseline and post‐intervention had to be ≥ 30%.

Missing data were deemed to be missing‐at‐random after performing sensitivity analysis of baseline and demographic variables, except anxiety levels at baseline were significantly greater in intervention participants with missing outcome measures at post‐intervention compared to those without. After performing best‐worst case analysis at post‐intervention and 3‐months post‐intervention, multiple imputation was considered the optimal approach (Gewandter et al. 2014; Hadjistavropoulos et al. 2022) in handling missing data for the secondary outcomes only for post‐intervention and 3 months post‐intervention, which included the BPI‐SF, BIOH, DASS‐21 and PSEQ and their subscales, which were all continuous. Multiple imputation was achieved through chained equations, where up to 10 iterations for each variable were specified, but only 5 were needed. Multiple imputation was applied to data simultaneously for the intervention and control groups. Outcome values at baseline, which were treated as numerical covariates in the general linear model, were not imputed but were used to enable imputation of missing values at post‐intervention and 3 months post‐intervention. Changes in PGIC, use of pain medication and other pain treatment, and occurrence of adverse events were calculated by complete case analysis and reported descriptively.

## 3 Results

### 3.1 Demographic and Clinical Characteristics

One‐hundred‐forty‐seven individuals were screened using the online form from January 2024 to March 2024 (refer to Figure 1 for CONSORT flow chart). One‐hundred‐twenty‐six individuals met the eligibility criteria and were contacted via email to be recruited to the study. Seventy‐two individuals consented, completed baseline measures and were randomised into either the control or the intervention group. The mean (SD) age of participants was 37.2 years (10.3), with 86% identified as female. Participant demographic characteristics are summarised in Table 1. There were 36 participants allocated to each group. Two participants in the control group requested withdrawal from the study after allocation, so their baseline results were excluded from analysis.

**TABLE 1:** Participant demographics and baseline measures.

	Control (*n* = 34)	Intervention (*n* = 36)
**Demographics**		
Mean age in years (SD)	36.9 (11.3)	37.4 (9.5)
Gender (%)		
Man/male	3 (8.8)	1 (2.8)
Woman/female	27 (79.4)	33 (91.7)
Non‐binary	0 (0)	2 (5.6)
Prefer not to answer	3 (8.8)	0 (0)
Genderfluid	1 (2.9)	0 (0)
Relationship status (%)		
Single/never married	19 (55.9)	15 (41.7)
Married/de facto	12 (35.3)	14 (38.9)
Separated/divorced	3 (8.8)	7 (19.4)
Pregnant (%)		
Yes	0 (0)	1 (2.8)
No	34 (100)	35 (97.2)
Highest education level (%)		
High school or less	7 (20.6)	2 (5.6)
Certificate/diploma	7 (20.6)	11 (30.6)
University	20 (58.8)	23 (63.9)
Employment status (%)		
Full time	8 (23.5)	10 (27.8)
Part time	11 (32.4)	12 (33.3)
Unemployed	5 (14.7)	3 (8.3)
Registered disability	7 (20.6)	5 (13.9)
Retired	1 (2.9)	0 (0)
Combination of above	2 (5.8)^a^	6 (16.7)^b^
**Baseline measures, mean (SD)**		
BPI‐SF pain intensity (0–10)^d^		
Worst	6.35 (1.82)	6.11 (1.47)
Least	2.56 (1.80)	2.61 (1.64)
Average	4.62 (1.74)	4.50 (1.28)
Right now	3.91 (2.30)	4.69 (1.82)
BPI‐SF pain interference (0–10)^d^	5.00 (2.45)	5.46 (2.10)
BIOH (0–360)^d^	233.00 (42.71)	235.61 (38.60)
DASS (0–42)^d^		
Stress	14.12 (6.32)^c^	17.83 (7.91)^c^
Anxiety	11.18 (8.37)	12.83 (8.01)
Depression	8.82 (8.66)	11.44 (8.55)
PSEQ (0–60)	28.24 (13.84)	27.86 (13.43)

Abbreviations: BIOH, Bristol Impact of Hypermobility; BPI‐SF, Brief Pain Inventory—Short Form; DASS, Depression Anxiety and Stress Scale; PSEQ, Pain Self‐Efficacy Questionnaire; SD, standard deviation.

^a^ 1 part‐time and registered disability and 1 retired and registered disability.

^b^ 3 part‐time and registered disability and 3 unemployed and registered disability.

^c^ Statistically significant, *p* < 0.05.

^d^ Higher score indicates worse outcomes.

At baseline, there were no differences in baseline characteristics between groups except for the DASS Stress score. The intervention group had higher stress levels (mean 17.8, SD 7.9) than the control group (mean 14.1, SD 6.3) (Table 1).

### 3.2 Primary Outcomes: Feasibility, Acceptability and Appropriateness

#### 3.2.1 FIM, AIM and IAM

**FIGURE 2:**
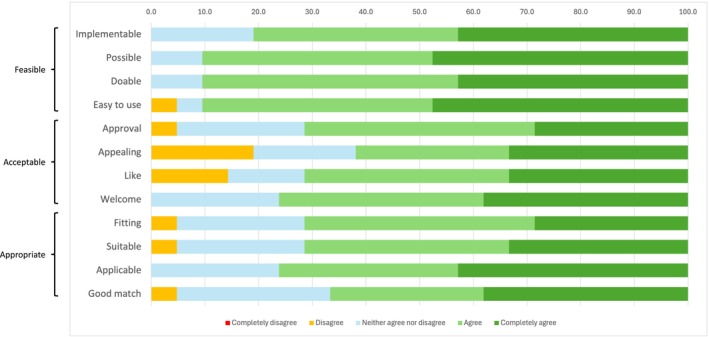
Feasibility of Intervention Measure (FIM), Acceptability of Intervention Measure (AIM) and Intervention Appropriateness Measure (IAM) results (*n* = 21).

Results for primary outcomes of feasibility, acceptability and appropriateness using the FIM, AIM and IAM, respectively, are shown in Figure 2. For the FIM, 81%–91% agreed or completely agreed that the HOPE program was feasible, and the mean score (SD) was 4.3/5 (0.71). For the AIM, 62%–76% agreed or completely agreed that the program was acceptable, with a mean score of 3.9/5 (0.96). The IAM showed that 67%–76% agreed or completely agreed that the program was appropriate, with a mean score of 4.0/5 (0.87).

#### 3.2.2 Rates of Completion of the Outcome Measures

All participants completed demographic characteristics and outcome measures at baseline.

At post‐intervention, 30 (88%) participants in the control group and 21 (58%) in the intervention group completed all measures. There was one incomplete questionnaire in each group. We attempted to contact these participants, but there was no response. The participant in the control group only completed change in use of pain medication and/or other pain treatment questions, while the intervention group participant only completed change in use of pain medication and/or other pain treatment questions and the BPI‐SF. The rest of the participants did not complete the FIM, AIM and IAM at post‐intervention (Figure 1).

At 3 months post‐intervention, 32 (94%) participants in the control group and 23 (64%) in the intervention group completed their outcome measures (Figure 1).

#### 3.2.3 Rates of Completion of the Intervention

Program completion rates in the intervention group are presented in Figure 1. We attempted to contact all participants who did not start the program, and two participants responded. Both suffered a medical episode prior to their intervention start date and did not feel well enough to take part.

#### 3.2.4 Website Analytics

Website analytics showed that the top 10 most visited pages were those that related to what the conditions were, why they experienced pain and how to develop a pain action plan. The number of visits and average time spent on each respective page are reported in Table 2. The number of views does not match the number of participants as each person could view each module multiple times. We did not track individual participant's website behaviour for privacy reasons.

**TABLE 2:** Website analytics—Top 10 pages visited (*n* = 29).

Page visited^a^	Number of visits	Average engagement time (s)
Module 3: What are hEDS and HSD?	94	132
Module 2: What are the different mechanisms of pain?	82	55
Module 1: Why am I in pain?	80	61
Module 6: How do I develop an action plan for my pain?	64	33
Module 7: How do I identify painful triggers and develop my own pain preventative strategies?	64	29
Module 5: How do I advocate for myself and communicate well with my healthcare team?	61	43
Module 4: Who and when do I seek help for my pain and hEDS/HSD?	59	25
Module 8: How does exercise, posture and movement affect my pain?	55	73
Module 9: How can I use external aids and modifications to help with my pain?	53	25
Module 2 checkpoint: Case example (Beth part 1)	50	71

^a^ Each module is made up of multiple pages. For example, Module 1 consisted of 13 pages.

### 3.3 Secondary Outcomes

Results of the effectiveness of the intervention are shown in Tables 3 and 4. At post‐intervention (Table 3), the DASS Stress score remained significantly higher in the intervention group (β = −4.79, 85% CI −9.05 to −0.54, *p* = 0.11) as it was at baseline; however, this difference was not maintained at 3 months post‐intervention. No effect of the intervention was found post‐intervention in BPI‐SF, BIOH, DASS Anxiety or DASS Depression, or PSEQ. At post‐intervention, according to the PGIC, more participants in the intervention group (33%) perceived a global improvement in their overall status than the control group (16%). Fewer participants in the intervention group reported a global worsening in their overall status (5%) than the control group (50%). Likewise, according to the change in pain medication and treatment usage, fewer participants in the intervention group reported increased pain medication usage and other pain treatment usage than those in the control group (9% and 29%, respectively, for both outcomes).

**TABLE 3:** Secondary outcomes at post‐intervention, T1 (post‐intervention vs. baseline).

Outcome measure	Control mean, T1 (SD), *n* = 34	Intervention mean, T1 (SD), *n* = 36	T1 baseline adjusted between‐group difference, β (85% CI)	*p*	Cohen's *d*
Brief Pain Inventory (BPI)—Pain intensity (0–10)^a^					
Worst pain	6.77 (1.69)	6.71 (2.28)	−0.08 (−0.68 to 0.53)	0.86	0.16
Least pain	3.55 (2.45)	3.11 (1.98)	0.48 (−0.18 to 1.15)	0.30	0.32
Average pain	5.17 (1.92)	5.05 (1.62)	0.06 (−0.50 to 0.61)	0.89	0.24
Pain right now	5.27 (2.39)	5.32 (2.64)	0.36 (−0.50 to 1.22)	0.54	0.14
BPI—Pain interference (0–10)^a^	5.77 (2.45)	5.60 (3.48)	0.41 (−0.54 to 1.35)	0.53	0.28
Bristol Impact of Hypermobility (0–360)^a^	242.18 (46.88)	236.64 (61.86)	7.69 (−9.54 to 24.93)	0.51	0.45
Depression Anxiety Stress Scale (0–42)^a^					
Depression	9.92 (11.02)	14.01 (11.76)	−2.01 (−5.22 to 1.19)	0.36	−0.41
Anxiety	10.74 (9.45)	13.15 (13.38)	−1.16 (−4.82 to 2.51)	0.64	0.13
Stress	13.95 (10.15)	18.78 (12.78)	−4.79 (−9.05 to −0.54)	0.11^b^	−0.37
Pain Self‐Efficacy Questionnaire (0–60)	27.48 (16.38)	28.16 (17.52)	−0.96 (−4.91 to 3.00)	0.73	−0.21
Patient global impression of change^c^, ^d^	Improved 5/30 (16.7%)	Improved 7/21 (33.3%)	NA	NA	NA
	No change 10/30 (33.3%)	No change 13/21 (61.9%)			
	Worsened 15 (50%)	Worsened 1/21 (4.8%)			
Change in use of pain medication^d^	Increased = 9/31 (29.0%)	Increased = 2/22 (9.1%)	NA	NA	NA
	Same = 21/31 (67.7%)	Same = 18/22 (81.8%)			
	Decreased = 1/31 (3.2%)	Decreased = 2/22 (9.1%)			
Change in use of other pain treatment (such as visits to GP, pain specialist or allied health therapists)^a^	Increased = 9/31 (29.0%)	Increased = 2/22 (9.1%)	NA	NA	NA
	Same = 22/31 (71.0%)	Same = 19/22 (86.4%)			
	Decreased = 0/31 (0%)	Decreased = 1/22 (4.5%)			

Abbreviation: NA, not applicable.

^a^ Higher score indicates worse outcomes.

^b^ Significant, *p* < 0.15.

^c^ Grouped into very much improved, much improved, minimally improved into ‘improved’ and minimally worse, much worse, very much worse into ‘worsened’.

^d^ Based on complete case analysis.

**TABLE 4:** Secondary outcomes at 3 months post‐intervention, T2 (3 months post‐intervention vs. baseline).

Outcome measure	Control mean, T2 (SD), *n* = 34	Intervention mean, T2 (SD), *n* = 36	T2 baseline adjusted between‐group difference, β (85% CI)	*p*	Cohen's *d*
BPI—Pain intensity (0–10)^a^					
Worst pain	6.50 (1.63)	5.28 (3.06)	1.12 (0.29 to 1.95)	0.06^b^	0.63
Least pain	3.54 (2.16)	2.93 (3.12)	0.65 (−0.36 to 1.66)	0.34	0.41
Average pain	4.66 (1.46)	4.31 (2.28)	0.29 (−0.34 to 0.92)	0.50	0.07
Pain right now	4.65 (2.04)	4.19 (2.94)	0.81 (−0.03 to 1.65)	0.17	0.25
BPI—Pain interference (0–10)^a^	4.19 (1.87)	4.09 (2.52)	0.31 (−0.40 to 1.02)	0.52	−0.11
Bristol impact of hypermobility (0–360)^a^	236.86 (42.92)	218.83 (55.98)	19.74 (4.98 to 34.51)	0.06^b^	0.32
Depression Anxiety Stress Scale (0–42)^a^					
Depression	10.21 (11.02)	11.73 (11.52)	0.44 (−2.51 to 3.39)	0.83	−0.01
Anxiety	9.53 (7.00)	10.66 (12.30)	0.03 (−2.74 to 2.80)	0.99	0.08
Stress	14.06 (9.04)	14.45 (12.00)	2.61 (−0.63 to 5.85)	0.24	0.08
Pain Self‐Efficacy Questionnaire (0–60)	25.96 (14.29)	30.21 (18.90)	−4.551 (−9.21 to 0.10)	0.16	−0.17
Patient global impression of change^c^, ^d^	Improved = 2/32 (6.3%)	Improved = 11/23 (47.8%)	NA	NA	NA
	No change = 13/32 (40.6%)	No change = 8/23 (34.8%)			
	Worsened = 17/32 (53.1%)	Worsened = 4/23 (17.4%)			
Change in use of pain medication^d^	Increased = 7/32 (21.9%)	Increased = 2/23 (8.7%)	NA	NA	NA
	Same = 24/32 (75.0%)	Same = 17/23 (73.9%)			
	Decreased = 1/32 (3.1%)	Decreased = 4/23 (17.4%)			
Change in use of other pain treatment (such as visits to GP, pain specialist or allied health therapists)^a^	Increased = 6/32 (18.8%)	Increased = 0/23 (0%)	NA	NA	NA
	Same = 24/32 (75.0%)	Same = 21/23 (91.3%)			
	Decreased = 2/32 (6.3%)	Decreased = 2/23 (8.7%)			

Abbreviation: NA, not applicable.

^a^ Higher score indicates worse outcomes.

^b^ Significant, *p* < 0.15.

^c^ Grouped into very much improved, much improved, minimally improved into ‘improved’ and minimally worse, much worse, very much worse into ‘worsened’.

^d^ Based on complete case analysis.

At 3 months post‐intervention (Table 4), the intervention group had significantly improved in their BPI worst pain with a moderate effect size (Cohen's *d* = 0.63) and in their BIOH with small effect size (Cohen's *d* = 0.32) compared to the control group. The number of adverse events based on a complete case analysis of participants who completed the adverse event outcome measure for each group was similar (Table 5). More participants in the intervention group reported a global improvement in their overall status (48%) than those in the control group (6%) at T2. Fewer participants in the intervention group reported worsening in their overall status (17% compared to 53% in the control group). Fewer participants in the intervention group reported an increase in pain medication usage than control participants (9% and 22%, respectively) as with other pain treatments (0% and 19%, respectively).

**TABLE 5:** Adverse event reporting based on complete case analysis of responses at post‐intervention (T1) and 3 months post‐intervention (T2).

Adverse event	Control, T1, *n* = 30, T2, *n* = 32	Intervention, T1, *n* = 21, T2, *n* = 23	Types of adverse event in intervention group
Life‐threatening			
T1	0	0	
T2	1	0	
Resulted in hospitalisation			
T1	2	2	Allergic reaction requiring hospitalisation for 1 day 1 visit for cardiac symptoms and 1 for pelvic pain and bleeding COVID infection requiring hospitalisation Knee surgery and injection for trochanteric bursitis Sinus surgery Anxiety seizures
T2	4	4	
Resulted in new persistent or significant disability			
T1	4	3	New spinal injury Broken ankle Infection leading to increased fatigue and weakness Knee surgery Anxiety seizures
T2	5	2	

#### 3.3.1 Minimal Clinically Important Difference

The median (range) expected change according to MCID was 46% (0%–89%) for worst pain intensity, least pain intensity, average pain intensity and pain intensity right now; 50% (9%–100%) for pain interference; 50% (10%–100%) for impact of hEDS/HSD on day‐to‐day life; 50% (0%–85%) for depression; 50% (0%–88%) for anxiety; 50% (5%–100%) for stress and 50% (0%–100%) for ability to perform in activities despite pain.

At post‐intervention, expected changes in outcomes were met or exceeded in intervention participants (*n* = 21): worst pain intensity in 2/21 (10%), least pain in 4/21 (19%), average pain intensity in 2/21 (10%), pain intensity right now in 2/21 (10%), pain interference in 1/21 (5%), impact of hEDS/HSD on day‐to‐day life in 0/21 (0%), depression in 3/21 (14%), anxiety in 5/21 (24%), stress in 5/21 (24%) and ability to perform in activities despite pain in 6/21 (29%).

At 3 months post‐intervention, expected changes in outcomes were met or exceeded in intervention participants (*n* = 23): worst pain intensity in 3/23 (13%), least pain in 5/23 (22%), average pain intensity in 2/23 (9%), pain intensity right now in 5/23 (22%), pain interference in 4/23 (17%), impact of hEDS/HSD on day‐to‐day life in 2/23 (9%), depression in 7/23 (30%), anxiety in 7/23 (30%), stress in 10/23 (43%) and ability to perform in activities despite pain in 8/23 (35%).

#### 3.3.2 Power Calculation

A post hoc power calculation (using clincalc.com) was performed based on the mean and SD of our BIOH outcome measure at 3 months post‐intervention. Two independent study groups using BIOH total score mean and SD with an alpha value of 0.15 gave us a post hoc power of 53%.

### 3.4 Changes Made to the Registered Trial Protocol

There were two changes made to our ANZCTR protocol. Firstly, there was a typo in our first protocol in the exclusion criteria. Severe depression was incorrectly written as > 20 using the PHQ‐9, and it was changed to the correct cut‐off of ^3^20. The second change was made to update our allocation blinding procedure. Initially, we did not have personnel to ensure allocation sequence blinding of the main author conducting the study. We were later able to secure personnel before recruitment and randomisation began. Both changes were accepted and updated in May 2024, 2 months after closing participant recruitment. These are reflected in the registry.

## 4 Discussion

The HOPE program is considered feasible, acceptable and appropriate by more than 60% of our hEDS/HSD participants. More than half of the intervention participants (58%) completed the program. The retention rate in both groups was more than half, but higher in the control than the intervention group (58% and 88%, respectively, at post‐intervention, 64% and 94% at 3 months post‐intervention). Participants in the HOPE program showed some improvements in worst pain intensity and impact of hypermobility.

Our secondary results showing preliminary effectiveness of the HOPE program are promising. Being a pilot study, we were not fully powered to establish program effectiveness. However, it was positive to see a moderate effect of the program on improving worst pain intensity, and a small effect on improving the impact of hypermobility 3 months post‐intervention, at a post hoc power of 53%. Additionally, at post‐intervention, the HOPE program seemed to prevent intervention participants' perception of their condition worsening, with 4.8% reporting worsening compared to 50% in the control group. At 3 months post‐intervention, a higher percentage of intervention participants reported improvements in their global impression of change (47.8% compared to 6.25% in the control group) and a lower percentage reported worsening (17.4% compared to 53% in the control group). The improvement in one of our functional outcome measures, that is Bristol Impact of Hypermobility, at only 3 months post‐intervention suggests that the HOPE program may have led to some purposeful behavioural change in our cohort's day‐to‐day lives. The infusion of self‐management skills using reflective tasks and allowing adequate time for the stages of behavioural change (e.g., opportunity, motivation and capacity) to make meaningful change may explain our findings (Lally et al. 2010; Ng et al. 2023). Although this preliminary finding demonstrated short‐term improvements to condition‐specific outcomes, future investigation through fully powered studies is required to determine the true effectiveness of the program and the variables leading to these potential improvements.

A feasibility study of an online pain education and exercise program for people with chronic musculoskeletal pain (ReabilitaDOR program, *n* = 54) that used the FIM, AIM and IAM noted a mean score of 4.5 across all three domains (Fioratti et al. 2022). Our scores were lower, especially our AIM (3.9/5). The AIM asks participants if they ‘approved’, ‘liked’, ‘welcomed’ or found the program ‘appealing’. We did not include open‐ended questions for participants to elaborate on the score they gave, but we postulate that our content may not have reached their expectations. The hEDS/HSD conditions are more complex, under‐recognised and poorly managed (Anderson and Lane 2021; Gensemer et al. 2021). Receiving a diagnosis can take up to 10 years (Halverson, Cao, et al. 2023); many would have spent varying amounts of time self‐investigating due to this lengthy delay. Social media has become widely used by this population to seek medical information (Farsi et al. 2022). In a qualitative survey of 24 hEDS participants, ‘Information seeking and vetting’ emerged as a reason they use social media with 89% using it to research their condition and find care (Halverson et al. 2024). Additionally, greater health literacy has been reported in those with higher education levels (Aljassim and Ostini 2020; Dinh et al. 2020), which is representative of our participants; majority had a university degree. Therefore, our participants may have preferred highly tailored information about their specific context with the condition (Bennett et al. 2022), rather than foundational information provided in the HOPE program. This was partly supported by our MCID results. In individual‐level analysis of change, most participants did not have any clinically worthwhile change in their outcomes, with up to only 29% reaching self‐determined MCID. The median MCID was about 50% across the secondary outcome measures, reflecting that participants were expecting the HOPE program to improve their pain, impact of hypermobility, emotional states and ability to perform in activities despite pain by 50%. These findings suggest that there are unmet healthcare needs and expectations among some with hEDS/HSD that require further exploration.

Furthermore, the lower levels of acceptability may have been due to the lack of social interaction in the program. People with hEDS/HSD have expressed synchronous healthcare practitioner interaction as being important (Halverson et al. 2024). A previous Delphi survey revealed that 69% of hEDS/HSD respondents felt it was important to have regular online contact with a trained healthcare professional and 73% felt that online discussion forums were important (Chew et al. 2024). While online pain management programs have shown to provide some cost savings (Dear et al. 2022; Pimm et al. 2020), including social interactions that involve extensive resources and costs. A fine balance between the inclusion of these to satisfy consumer needs and the long‐term self‐sustainability of the HOPE program is required, such as making these services optional (Dear et al. 2021) or partnering with tertiary‐level education centres or hospitals to provide them at no or low cost when the clinical and cost effectiveness of the programs is demonstrated (Dear et al. 2018; Smith et al. 2019).

To date, there are only two other published papers about online pain management programs for hEDS/HSD, both of which were single group, pre–post studies. One study evaluated a multidisciplinary group‐based synchronous telemedicine program called ‘EDS Living’ in North America (Knight et al. 2023) and the other study evaluated an asynchronous online mindfulness program in the United Kingdom (Lattimore and Harrison 2023). The former reported that their program met participant needs and improved their understanding of the condition, while the latter reported that online‐delivered mindfulness therapy resulted in improvements in quality of life. These two studies did not report feasibility measures, and only the latter reported dropout rates from baseline to post‐intervention of 52% (81/157) (Lattimore and Harrison 2023). Our study recorded a comparable dropout rate of 47% (15/32) in the intervention group. The findings from these two studies in combination with results from this HOPE pilot study inform the first steps in investigating and designing future hEDS/HSD‐specific online pain management programs. There were promising results from the two studies, but the authors did not report if stakeholder needs and context were considered and addressed while designing their interventions. Whilst our study incorporated stakeholder input before the design of the HOPE program (Chew et al. 2024), we did not involve stakeholders in the study design and implementation stages (Webber et al. 2022), which could be another possible reason for the high non‐completion and dropout rates (Slattery et al. 2020). Involving those with lived experience may improve user engagement, satisfaction, and usefulness of online interventions (Bennett et al. 2022; Elbers et al. 2021).

There were several further limitations in our study. Firstly, eligibility based on medical diagnosis of hEDS/HSD was self‐reported and it is possible that individuals without hEDS/HSD were included. Secondly, we had a larger dropout in the intervention group despite multiple attempts to follow up. We sent three email reminders to intervention participants throughout the 8 weeks and up to three email reminders to all participants regarding follow‐up surveys. Further studies may consider using other avenues to increase adherence (e.g., automated application software, routine check‐ins via phone or video conferencing). Lastly, the variability in our results suggests that we could have collected more demographic details, such as participant ethnicity, how long it took to receive their diagnoses, baseline health literacy and website analytics about individual usage and engagement. These may help with modifications to the program to serve different demographics or discern who the program may be better suited for, so that future studies can focus on targeted populations.

We have several recommendations for improvements and future fully powered trials of the HOPE program, or for researchers wanting to develop similar interventions for hEDS/HSD. We recommend including trained healthcare professional contact such as one‐on‐one/small group sessions or clinician‐moderated online discussion forums as part of the program (Chew et al. 2024). We suggest collecting more participant characteristics and website analytics so that interventions can be targeted at subgroups who would see the greatest benefit. Potentially, those who start with higher pain intensity and greater impact of hypermobility or who are newer to their diagnosis may see greater effectiveness. Based on our results, collecting post‐intervention outcomes at a later time point (e.g., 6, 9 or 12 months post) may be useful as our results showed greater differences at 3 months rather than immediate post‐intervention. Lastly, we recommend collecting additional outcome measures pertinent and meaningful to the hEDS/HSD population given their unique, multisystemic symptoms, such as fatigue (Clark et al. 2024).

In summary, this pilot study of the HOPE program determined that the program is feasible, acceptable and appropriate. Our high dropout rates and low program completion rates were comparable to other online intervention studies conducted in similar populations, suggesting that there are still areas of improvement for future trial design. Despite this being a pilot study, small to moderate improvements in the impact of hypermobility and worst pain intensity were observed. These preliminary effectiveness findings warrant further investigation with a larger, fully powered hEDS/HSD cohort. Future iterations of the HOPE program could consider including regular online healthcare provider contact and online forums, as well as moving across different platforms for greater accessibility and improved acceptance.

## Author Contributions

This study was designed and conducted by Min Tze Chew, Cliffton Chan, Emre Ilhan, Leslie L. Nicholson and Sarah Kobayashi. Data were analysed by Min Tze Chew, Emre Ilhan and Cliffton Chan, and results were critically examined by all authors. All authors were involved in writing the manuscript. All authors have approved the final versions of this manuscript and agree to be accountable for all aspects of this work.

## Supporting information


**Appendix S1.** CONSORT checklist.

**Appendix S2.** HOPE program content summary.
