# Supplementary material for: HOPE for Hypermobile Ehlers–Danlos Syndrome (hEDS) and Hypermobility Spectrum Disorder (HSD)—A Pilot Randomised Controlled Trial of Feasibility, Acceptability and Appropriateness
Source: Eur J Pain. 2025 Apr 29;29(6):e70030. doi: 10.1002/ejp.70030 (PMC12038781; doi:10.1002/ejp.70030)

Supplement B: Description of the HOPE program

**Module title**	**Description**
1: Why am I in pain?	In the first module, content explores the following concepts: *"What* is pain; *why* pain exists; and *how* do we experience pain?". Checkpoint: There are reflective questions prompting participants to consider their own pain experiences and what biopsychosocial factors might be contributing to their pain.
2: What are the different mechanisms of pain?	Content explores *"how* we experience pain", the different pain mechanisms and a case example to expound on these.
3: What are hEDS and HSD?	In this module, participants are presented with information about hEDS and HSD, how they are diagnosed and their signs and symptoms.
4: Who and when do I seek help for my pain and hEDS/HSD?	Participants are introduced to some healthcare professionals who can help them on their journey with hEDS/HSD. Checkpoint: There are reflective questions to help participants think about who they may need on their multidisciplinary team.
5: How do I advocate for myself and communicate well with my healthcare team?	Participants are presented with content discussing how they can advocate for themselves and improve communication to maximise therapeutic alliance with their healthcare team. Checkpoint: Participants are asked to think back to challenging and/or positive interactions with their healthcare team, and to think about any suggestions from the module that they may want to adopt to improve their therapeutic alliance with their healthcare team.
6: How do I develop an action plan for my pain?	Participants are introduced to the concept of a pain action plan, and given suggestions on how they can build their own individualized action plan. There are case examples to facilitate further thinking and to show practical examples of how pain action plans can be implemented. Checkpoint: Participants are prompted to think back on their own flare-up episodes, how they navigated it, and if and how an action plan would have changed it. They are also prompted to think of building their own action plan, or to revisit their existing action plan and consider if they can improve on it.
7: How do I identify painful triggers and develop my own pain preventative strategies?	In module 7, participants are presented with some common pain triggers and strategies to help prevent them. Checkpoint: Participants are asked to reflect on some of their own pain triggers, using the biopsychosocial model. They are then encouraged to think about what strategies they can adopt to address their pain triggers.

8: How does exercise, posture and movement affect my pain?	In this module, participants are presented with information on how exercise, posture and movement retraining can be beneficial for people with hEDS and HSD.<br>Checkpoint: Participants are asked to try and identify possible postures or movements that tend to aggravate their pain, what exercise/posture/movement to they avoid/fear, and if there are any emotions or thoughts related to what they avoid/fear. They are also prompted to think about what exercises or activities they think may be helpful for them at their current stage.
9: How can I use external aids and modifications to help with my pain?	Participants are provided with suggestions and examples of some modifications that people use for their pain or for other hEDS/HSD symptoms that indirectly affect pain.<br>Checkpoint: Participants are asked to reflect back on the information in the module, and if they require aids or modifications, and to think about how to implement those or who they may need to go to for more information or assistance.
10: What medical managements are available for my pain?	Sometimes, people with hEDS/HSD may need more support through medical devices or medication. In module 10, participants are shown common pain medication, supplements, medical devices and surgical options for their pain.
11: What does pelvic health and neurodiversity have to do with pain in hEDS/HSD?	In module 11, participants are provided with information about how pelvic health and neurodiversity affect pain.
12: Summary	In this last module, participants are given the summary of take-home messages from each module. They are also encouraged to revisit any module they want.

Screenshots of the HOPE website

a) Example of each module checklist page which is automatically checked off when participants complete each section

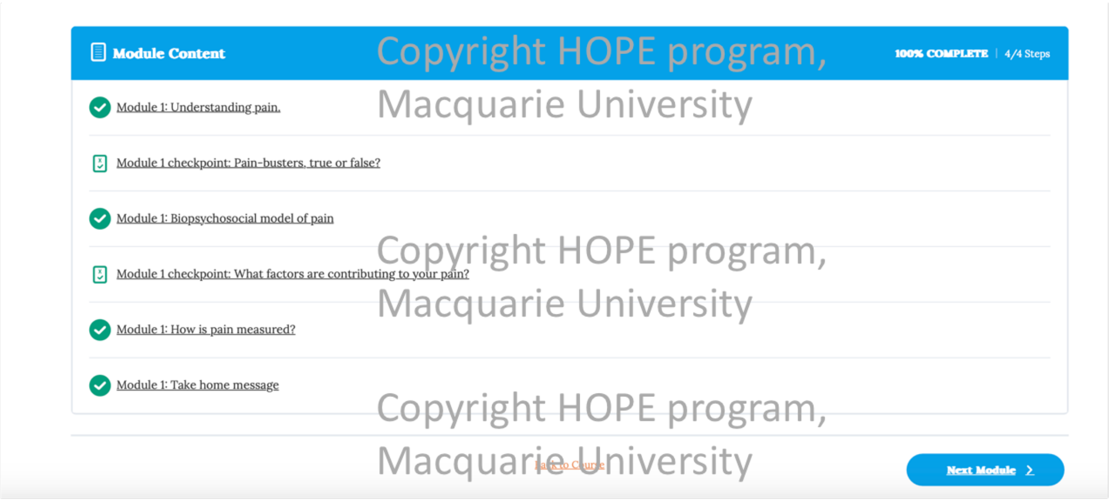

b) Information is presented in smaller chunks on each page, with graphics/pictures

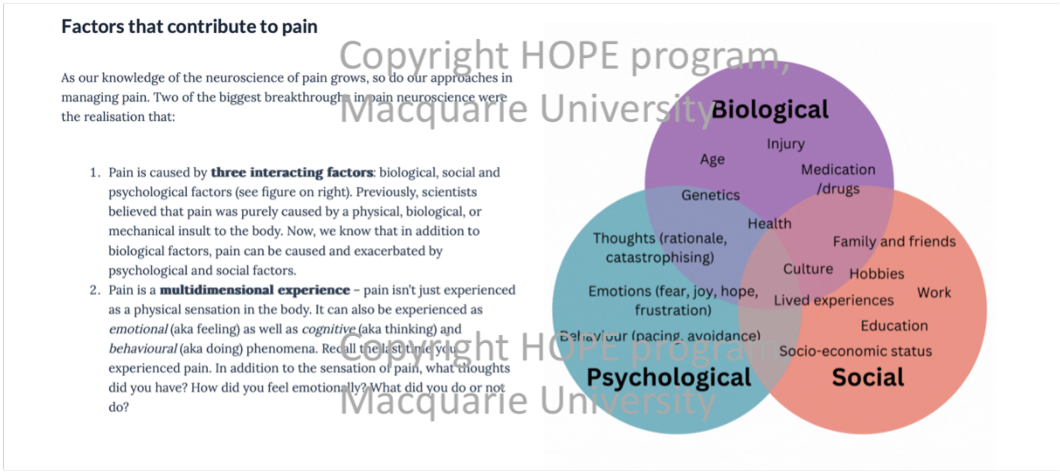

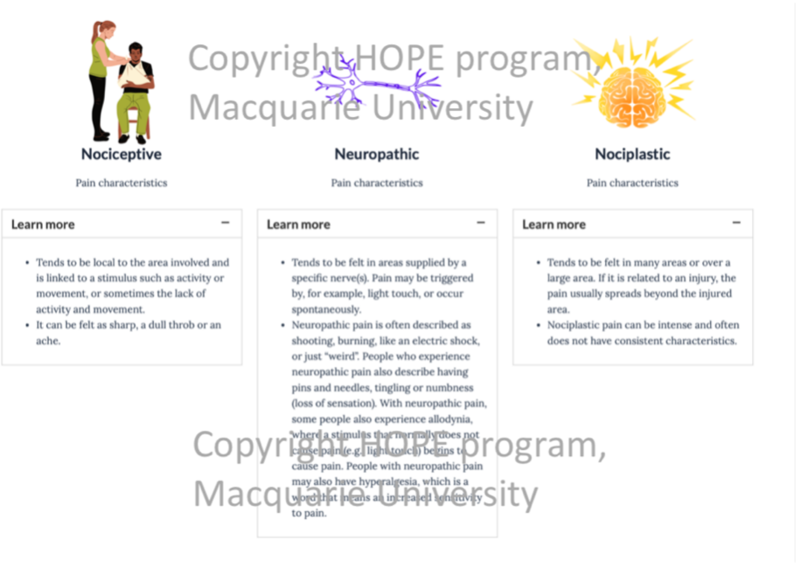

c) Photos and videos of exercise examples

2. Pay attention to how you breathe. Pain, thoughts and emotions change the way you breathe. It makes you breathe shallow and use an upper chest and neck breathing pattern rather than a deep, diaphragmatic breathing pattern. This shallow breathing pattern causes some muscles in your neck, chest and trunk to overwork, which may lead to pain. Checking your breathing through the day and resetting your breathing through the day can be a simple yet powerful way to reduce pain. Click the 'play' button to watch a video example of breathing exercises.

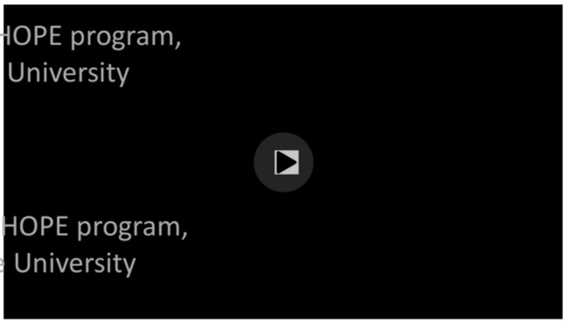

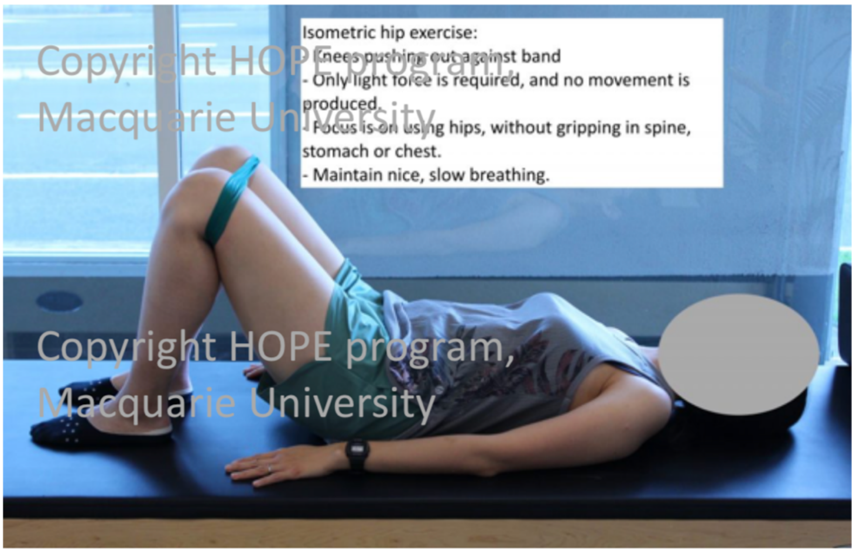

Supplement: Supplementary file 2 — Appendix S2. HOPE program content summary. [file EJP-29-0-s001.pdf]
